# Klinisches Screening für HNO-Ärzte bei potenziell somatosensorischem Tinnitus aurium: Der SOMASENSO-Check zur Identifikation von manualmedizinisch relevanten Funktionsstörungen

**DOI:** 10.1007/s00106-025-01603-6

**Published:** 2025-03-27

**Authors:** Anett Reißhauer, Isabelle Hoffmann, Birgit Mazurek, Norman Best, Max E. Liebl

**Affiliations:** 1 Arbeitsbereich Physikalische Medizin, Charité – Universitätsmedizin Berlin, corporate member of Freie Universität Berlin and Humboldt-Universität zu Berlin, Charitéplatz 1, 10117 Berlin, Deutschland; 2 Tinnituszentrum, Charité – Universitätsmedizin Berlin, corporate member of Freie Universität Berlin and Humboldt-Universität zu Berlin, Charitéplatz 1, 10117 Berlin, Deutschland; 3 Zentrum für Physikalische und Rehabilitative Medizin, Sophien- und Hufeland-Klinikum Weimar, Henry-van-de-Velde-Straße 2, 99425 Weimar, Deutschland; 4 Arbeitsbereich Physikalische Medizin, Charité – Universitätsmedizin Berlin, Charitéplatz 1, 10117 Berlin, Deutschland

**Keywords:** Komplementäre Therapien, Ganzheitliche Gesundheitsversorgung, Physikalische Therapiemodalitäten, Somatosensorische Erkrankungen, Manuelle Therapie, Complementary therapies, Holistic health, Physical therapy modalities, Somatosensory disorders, Manual therapy

## Abstract

**Hintergrund:**

Ein somatosensorischer Tinnitus ist mit Funktionsstörungen der Halswirbelsäulenregion und/oder der Kiefergelenkregion vergesellschaftet. Hier kann die manuelle Medizin hilfreich sein. Die diagnostischen Kriterien für einen somatosensorischen Tinnitus sind jedoch nur z. T. aus der spezifischen Anamnese abzuleiten, teils sind darüber hinaus klinisch-manuelle Untersuchungstechniken notwendig. Ziel der Arbeit war es, ein pragmatisches, in der HNO-Praxis im Sitzen durchführbares Screeninginstrument auf somatosensorischen Tinnitus zu entwerfen.

**Material und Methoden:**

In einem modifizierten Delphi-Verfahren identifizierte eine Gruppe aus Manualmedizinern und HNO-Ärzten geeignete manualmedizinische Verfahren und bewertete sie auf ihre Durchführbarkeit in der HNO-Arztpraxis.

**Ergebnisse:**

Der SOMASENSO-Check ist eine pragmatische Untersuchungshilfe, die HNO-Ärzten die Identifizierung potenzieller somatosensorischer Tinnituspatienten erleichtert.

**Schlussfolgerung:**

Die technische Schwierigkeit und mangelnde Test-Retest-Reliabilität vieler manueller segmentaler Funktionstests führte zu einer Fokussierung auf die Detektion von Bewegungsstörung, Schmerz und Tinnitusmodulation.

## Abstract (en)

**Background:**

Somatosensory tinnitus is associated with functional disorders of the cervical spine and/or temporomandibular joint region. Manual medicine can be helpful here. However, the diagnostic criteria for somatosensory tinnitus can only partly be derived from the specific anamnesis; in some cases, clinical manual examination techniques are also necessary. The aim of this work was to design a pragmatic screening instrument for somatosensory tinnitus that can be carried out in the ENT practice with a seated patient.

**Materials and methods:**

In a modified Delphi procedure, a group of manual medicine specialists and otorhinolaryngologists identified suitable manual medicine procedures and evaluated them for their feasibility in the ENT practice.

**Results:**

The SOMASENSO check is a pragmatic examination tool that makes it easier for otorhinolaryngologists to identify potential somatosensory tinnitus patients.

**Conclusion:**

The technical difficulty and lack of test–retest reliability of many manual segmental function tests has led to a focus on the detection of movement disorders, pain, and tinnitus modulation.

Somatosensorischer Tinnitus ist mit Funktionsstörungen der Halswirbelsäulenregion und/oder der Kiefergelenkregion vergesellschaftet. Hier kann die manuelle Medizin hilfreich sein. Doch welche Patientinnen und Patienten soll eine HNO-Praxis zum Manualmediziner überweisen? Es ist ein niedrigschwelliges Screening nötig, welches in der HNO-Praxis sicher und sensitiv angewendet werden kann.

## Somatosensorischer Tinnitus

Somatosensorischer Tinnitus aurium (SST) ist eine klinisch bedeutsame Untergruppe des chronischen subjektiven Tinnitus. Die Pathogenese ist dabei noch unvollständig verstanden. Klar ist jedoch, dass es eine Verschaltung zwischen somatosensorischem und auditorischem System gibt: So wird etwa der Nucleus cochlearis dorsalis durch somatosensorische Einflüsse disinhibiert, und klinisch zeigt sich in manchen Fällen eine Modulation der Geräusche durch Bewegungen von Kopf, Halswirbelsäulen(HWS)-Region oder Augen, zudem ist – vice versa – bei Funktionsstörungen des Kiefergelenks die Prävalenz von Tinnitus deutlich erhöht [1–3].

Ein internationaler Delphi-Prozess erarbeitete Kriterien, die auf einen starken somatosensorischen Einfluss auf das Ohrgeräusch hindeuten [4]. Hierbei sind besonders anamnestische Angaben zur Geräuschmodulation durch Bewegungen relevant.

Auch wurden speziell im Bereich der Halswirbelsäulen- und Kiefergelenke sowie der umgebenden Muskulatur entsprechende segmentale Gelenkfunktionsstörungen und Muskeltriggerpunkte [5] identifiziert, sodass teils auch von einem zervikogenen somatosensorischen Tinnitus gesprochen wird [6]. Eine randomisierte kontrollierte Studie (RCT) postulierte die Bedeutung spezifischer nuchaler Streckmuskeln (v. a. M. semispinalis capitis und M. splenius capitis) sowohl für die Diagnostik als auch für die manuelle Therapie [7]. Eine Behandlung von „physikalischen“ Beschwerden wurde auch in der aktuellen S3-Leitlinie aufgenommen [8]. Die diagnostischen Kriterien für einen somatosensorischen Tinnitus sollten daher eine

- somatosensorische Modulation,
- spezifische Tinnituscharakteristika, sowie
- spezifische Begleitsymptome

umfassen [4]. Nur z. T. sind diese jedoch aus der spezifischen Anamnese abzuleiten, die körperliche Untersuchung kann ebenso wegweisend sein, mit einem SST vergesellschaftete Störungen der HWS und Kiefergelenkregion zu detektieren [4]. Teils sind darüber hinaus Untersuchungstechniken notwendig, die manualmedizinische Grundkenntnisse erfordern. Eine Arbeitsgruppe aus Regensburg schlägt ein ausführliches manualmedizinisches Untersuchungsprogramm vor [9]. In Praxi erfolgt die Erstvorstellung von Patientinnen und Patienten mit Ohrgeräusch häufig jedoch bei Hals-Nasen-Ohren-Ärzten ohne manualmedizinische Zusatzausbildung. Bei der bekannten Häufigkeit der Diagnose „Tinnitus aurium“ und der Zahl der Neuerkrankungen ist eine regelhafte Überweisung zur manualmedizinischen Exploration jedoch unnötig und unökonomisch.

## Pragmatisches Screening

Daher ist ein an der HNO-ärztlichen Praxis orientiertes kombiniertes anamnestisches und manualmedizinisches Screening sinnvoll, um mit möglichst hoher Sensitivität die betreffenden Patientinnen und Patienten mit SST zu identifizieren, nachrangig aber diejenigen ohne Hinweis auf SST auszuschließen.

Das Ohrgeräusch ist als potenziell somatosensorisch modulierbar einzustufen, wenn sich klare Hinweise ergeben (a) bereits aus den von Michiels zusammengetragenen, überwiegend anamnestischen Kriterien, oder (b) aus den pragmatischen klinischen Untersuchungen des hier vorgestellten SOMASENSO-Checks.

Hierzu entwickelte eine interdisziplinäre Expertenrunde aus Manualmedizinern (Fachärzten für Physikalische und Rehabilitative Medizin mit Zusatzweiterbildung Manuelle Medizin) und HNO-Ärzten – die Autoren dieses Beitrages – in einem modifizierten Delphi-Verfahren zwischen 06/2023 und 04/2024 eine einfache klinische Untersuchungshilfe.

Bedingungen für diese Untersuchungshilfe waren,

- den praktischen Erwägungen der Anwendbarkeit in einer HNO-Praxis genügen können und
- gleichzeitig die klinische Untersuchung auf die Kriterien zur Untersuchung der somatosensorischen Modulation des Tinnitus zu beinhalten.

Patienten, die auf diese Weise detektiert werden, können im weiteren Verlauf an Manualmediziner zur weiteren differenzierten Untersuchung und Behandlung überwiesen werden. Dieser Beitrag beinhaltet keine Studien an Menschen oder Tieren.

## Untersuchungshilfe „SOMASENSO-Check“

**Abb. 1:**
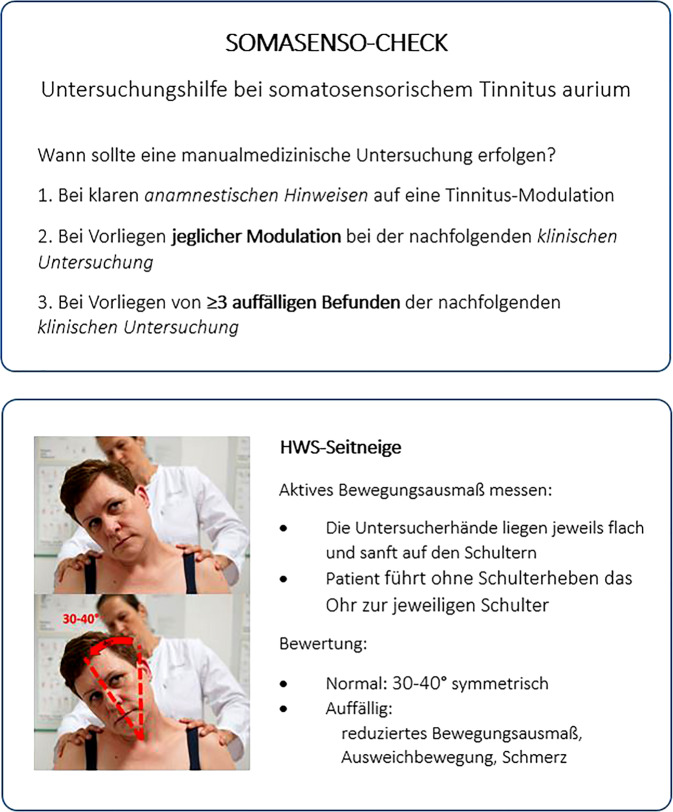
SOMASENSO-CHECK-Algorithmus; HWS-Seitneige. (© A. Reißhauer)

**Abb. 2:**
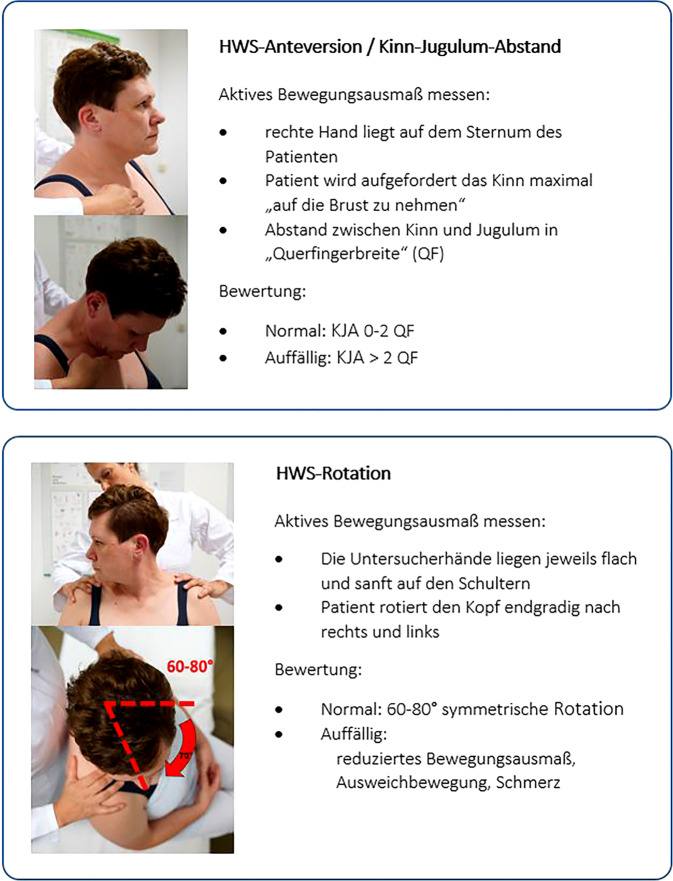
Kinn-Jugulum-Abstand und Halswirbelsäulen(HWS)-Rotation. (© A. Reißhauer)

Die Abb. 1, 2, 3 und 4 stellen die ausgewählten Screeninguntersuchungen und die diagnostischen Kriterien dar.

**Abb. 3:**
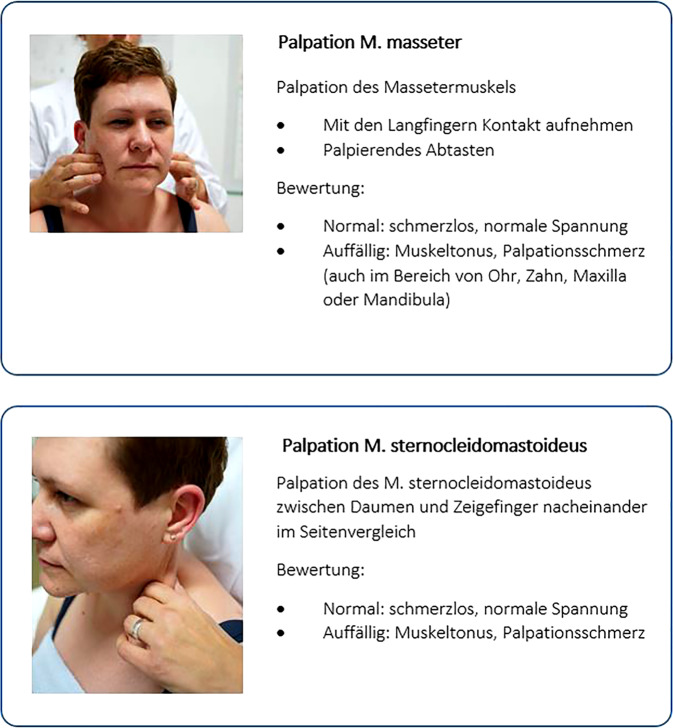
Muskelpalpation von M. masseter und M. sternocleidomastoideus. (© A. Reißhauer)

**Abb. 4:**
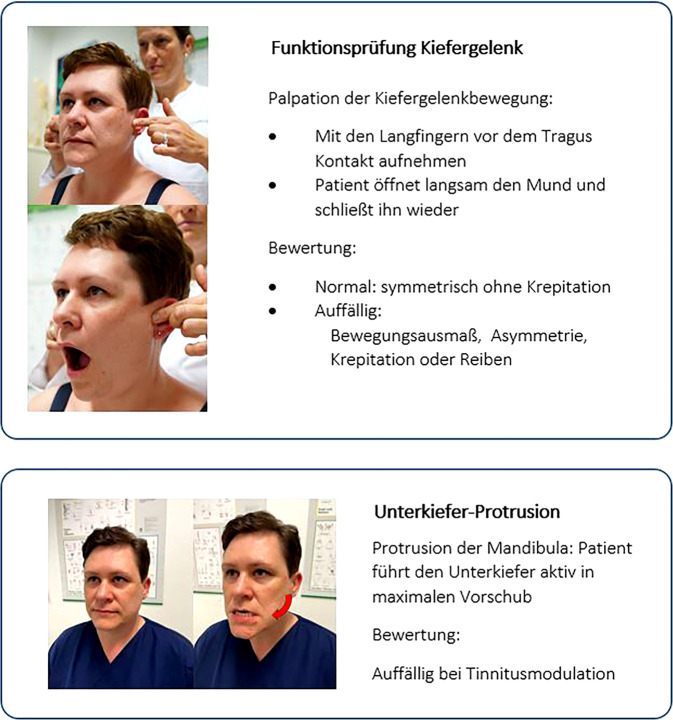
Funktionsprüfung von Kiefergelenk und Unterkieferprotrusion. (© A. Reißhauer)

## Detektion von Tinnitusmodulation, Bewegungsstörung und Schmerz

Zu den vorgeschlagenen Testverfahren: Jede wie auch immer geartete Modulation des Ohrgeräuschs durch Bewegung oder Berührung bei der Untersuchung muss dazu führen, dass zumindest der Verdacht besteht, den Tinnitus beeinflussen zu können, und stellt daher aus Sicht der Autoren eine Indikation zur Vorstellung beim manualmedizinisch versierten Untersucher dar. Insbesondere die Mandibulaprotrusion scheint hier – empirisch – eine sensitive Untersuchung zu sein.

Da Bewegungsstörungen und Schmerzen in der kraniomandibulären Region und der HWS-Region sehr häufig sind, sollte – bei Nichtvorliegen einer Tinnitusmodulation – das Ausmaß dieser Störungen bei der Bewertung relevant sein. Daher schlagen die Autoren vor, die Einschränkung des Bewegungsumfangs der Halswirbelsäule, die Muskelpalpation von M. masseter und sternocleidomastoideus sowie die Funktionsprüfung des Kiefergelenks erst mit 3 oder mehr auffälligen Tests der dargestellten klinischen Untersuchungen als auffälliges Screening zu werten.

Segmentale Tests wurden bewusst nicht vorschlagen. Derlien et al. zeigten, dass schmerzdetektierende Tests in ausreichendem Maße reliabel sind [10]. Für gezielte manualmedizinisch-osteopathische Untersuchungstechniken, insbesondere für segmentale Untersuchungstechniken, ist dies nicht ohne Weiteres realisierbar. In einer weiteren Studie von Geipel et al. zeigte sich ebenfalls, dass selbst für versierte Untersucher die Wiederholbarkeit gezielter bzw. segmentaler manualmedizinisch-osteopathischer Tests allenfalls für regionale Fragestellungen erzielbar ist [11]. In diesem konkreten Fall ließ sich keine akzeptable Reliabilität für segmentale Untersuchung der oberen HWS-Abschnitte erreichen. Dies änderte sich in dem Moment, als man global die „Kopfgelenkstörung“ als Outcome- bzw. Untersuchungsparameter definierte.

Da die Untersuchung im Sitzen stattfinden soll, kommt eine orientierende Palpation des Zwerchfells als Screening ebenso nicht infrage, auch wenn diese aus manualmedizinischer Sicht wertvoll wäre.

Von einigen Autoren wird postuliert, dass insbesondere eine Irritation des M. splenius capitis und M. semispinalis zu einer Tinnitusmodulation beitragen könne [7]. Die letztlich noch offene Frage, ob diese langfaserigen, bewegungssichernden Muskeln am SST beteiligt sind oder nicht doch eher die darunterliegenden, für die Feinmotorik des Kopfs zuständigen, sog. kurzen Nackenstrecker zwischen dem Okziput und den Segmenten C1 und C2, ließe sich durch eine Palpation der ganzen subokzipitalen Gelenkregion überwinden. Technisch würde man hier jedoch im Liegen palpieren, um nicht durch die Vorspannung die Palpationsbefunde zu behindern. Im Sitzen wäre es denkbar, die Palpation der Subokzipitalregion im Pinzettengriff mit am Untersucher abgestützten Kopf vorzunehmen. Diese Abstützreaktion ist wichtig, da sonst keine valide Palpation erfolgen kann, weil die langstreckige Muskulatur, die über den zu palpierenden Muskelschichten liegt, aktiviert ist, um die Haltung und Stellung des Kopfs zu sichern, die bei fehlender Abstützung einer Retroflexion der HWS entspricht. Dies halten wir in der Screeningsituation der HNO-Praxis jedoch nicht für zielführend, sodass letztlich auf diese Palpationstechnik verzichtet wurde.

Die technische Schwierigkeit und mangelnde Test-Retest-Reliabilität vieler manueller segmentaler Funktionstests führte zu einer Fokussierung auf die Detektion von Bewegungsstörung, Schmerz und Tinnitusmodulation.

Weiterführende Untersuchungen bei vorhandener manualmedizinischer Expertise könnten zusätzlich zu den genannten ausgeschlossenen Verfahren dann z. B. eine posturale Befundung, dynamische Bewegungsanalyse, differenzierte Muskelpalpation oder segmentale Gelenkfunktionsuntersuchungen der HWS und der Kopfgelenkregion sein. Aber auch Nervendehnungstests, Provokationstests und die detaillierte Untersuchung des Temporomandibulargelenks einschließlich dessen übergreifender Muskelgruppen bis hin zur enoralen Palpation der Pterygoideusgruppe sind in spezifischeren Diagnostiksituationen angezeigt.

Screeningtests werden eingesetzt, um Krankheiten möglichst früh zu identifizieren und dagegen einzugreifen mit dem Ziel, die Morbidität zu verringern. Screeningtests liefern keine endgültige Diagnose, und Screeningtests „beweisen“ nicht, dass eine Person eine Krankheit hat, sondern liefern einen Verdacht. Auf einen positiven initialen Screeningtest folgt ein weiterer diagnostischer Test, der den Verdacht erhärten soll, in diesem Fall die Vorstellung beim Manualmediziner. Beweisende Test gibt es derzeit nicht. Die somatosensorische Komponente bzw. der SST ist eine *Diagnosis ex juvantibus*, denn erst die positive Beeinflussung der Symptomatik durch manuelle bzw. physikalische Therapien [12] lässt eine hinreichend sichere Aussage zu.

Eine Sensitivitätsanalyse des SOMASENSO-Check wird aktuell geplant.

## Fazit für die Praxis

- Der SOMASENSO-Check fasst wichtige Untersuchungstechniken zusammen, um einen somatosensorischen Tinnitus aurium niedrigschwellig zu identifizieren.
- Die Auswahl der Untersuchungstechniken ist auf die Gegebenheiten einer HNO-Praxis pragmatisch zugeschnitten.
- Zusammen mit einer spezifischen Anamnese ergibt sich ein Screeninginstrument, das entsprechende Patientinnen und Patientin „herausfischen“ kann und einer manualmedizinischen Diagnostik und Behandlung zuführen soll.

## Data Availability

Die erhobenen Datensätze können auf begründete Anfrage in anonymisierter Form beim korrespondierenden Autor angefordert werden.
